# Schizophrenia in Covid-19 crisis : Is it a mortality risk factor ?

**DOI:** 10.1192/j.eurpsy.2022.1303

**Published:** 2022-09-01

**Authors:** K. Nourchene, E. Khelifa, B. Abassi, S. Ben Aissa, O. Maatouk, I. Bouguerra, L. Mnif

**Affiliations:** 1 Razi hospital, Pedopsychiatry, Tunis, Tunisia; 2 Razi hospital, Skolly, Tunis, Tunisia; 3 Razi Hospital, F Adult Psychiatry Department, Manouba, Tunisia; 4 Razi Hospital, Psychiatry Ibn Omran, Manouba, Tunisia; 5 Hôpital Razi, Psychiatry F, Manouba, Tunisia; 6 Errazi hospital-Mannouba, F, Mannouba, Tunisia

**Keywords:** Covid-19, schizophrénia, mortality

## Abstract

**Introduction:**

Patients with mental disorders mainly schizophrenia represent a vulnerable population. In Covid-19 pandemic situation, could schizophrenia be considered as a significant mortality risk factor ?

**Objectives:**

In this study, we aimed to explore the odds of significant COVID-19 mortality among schizophrenia patients

**Methods:**

Our literature review was based on the PubMed interface and adapted for 2 databases: Science Direct and Google Scholar using the following combination ( schizophrenia [MeSH terms]) AND (COVID-19, mortality[MeSH terms])

**Results:**

Our review included 4 population-based cohort studies covering the period from december 2019 to May 2021. The data showed increased mortality risk among individuals with schizophrenia who have had COVID-19. Indeed, this high rate of mortality maybe associated with multiple factors such as unhealthy lifestyle, low socioeconomic status and comorbidities as obesity, diabetes and cardiovascular conditions. The use of antipsychotics can be considered as a risk factor regarded its immunomodulatory effects. Furthermore, stigma and discrimination towards mental illnesses particularly schizophrenia might have contributed to a worse prognosis.

**Conclusions:**

Schizophrenia is a severe mental disorder ,associated with an increased high risk Covid-19. Thus, this population require enhanced preventive and disease management strategies.

**Disclosure:**

No significant relationships.

